# Iodinated Polyesters with Enhanced X-ray Contrast Properties for Biomedical Imaging

**DOI:** 10.1038/s41598-020-57720-5

**Published:** 2020-01-30

**Authors:** Timothy R. Lex, Beau R. Brummel, Mohamed F. Attia, Lauren N. Giambalvo, Kinsey G. Lee, Brooke A. Van Horn, Daniel. C. Whitehead, Frank Alexis

**Affiliations:** 1 Department of Chemistry, Clemson University, 467 Hunter Laboratories, Clemson, SC 29634 USA; 2 Department of Bioengineering, Clemson University, 301 Rhodes Research Center, Clemson, SC 29634 USA; 3 School of Biological Sciences and Engineering, Yachay Tech, San Miguel de Urcuquí, Ecuador; 4 Department of Chemistry and Biochemistry, College of Charleston, 66 George St., Charleston, SC 29414 USA

**Keywords:** Biomedical materials, Biomedical materials

## Abstract

Synthetic materials exhibiting contrast imaging properties have become vital to the field of biomedical imaging. However, polymeric biomaterials are lacking imaging contrast properties for deep tissue imaging. This report details the synthesis and characterization of a suite of aryl-iodo monomers, which were used to prepare iodinated polyesters using a pre-functionalization approach. Commercially available 4-iodo-phenylalanine or 4-iodobenzyl bromide served as the starting materials for the synthesis of three iodinated monomeric moieties (a modified lactide, morpholine-2,5-dione, and caprolactone), which under a tin-mediated ring-opening polymerization (ROP), generated their respective polyesters (PE) or poly(ester amides) (PEA). An increase in X-ray intensity of all synthesized iodine-containing polymers, in comparison to non-iodinated poly(lactic acid) (PLA), validated their functionality as radio-opaque materials. The iodinated-poly(lactic acid) (iPLA) material was visualized through varying thicknesses of chicken tissue, thus demonstrating its potenial as a radio-opaque biomaterial.

## Introduction

Medical imaging, a technique that provides structural visualization inside the body, aides in the study of specific morphological changes within living and nonliving systems^1,2^. One particular imaging modality, X-ray radiography, is frequently used as a diagnostic tool for non-invasive, *in vivo*, real-time examinations of three-dimensional opaque objects. X-ray imaging can be used to monitor response, degradation, and defects of biomedical devices^3^. Concerns over the long-term stability of prolonged or permanent implantable devices have led to the development of polyester-based materials due to their desirable properties (i.e. biocompatibility, biodegradability, and facile synthesis)^4–8^. The evolution of biodegradable polyester devices, like staples, stents, sutures, and implants, have had a significant impact on the biomedical field^4,9,10^. Perhaps the most noteworthy advantage is their ability to be degraded and excreted from the body, obviating the need for their removal or surgical revision. This can be vital in major surgical procedures such as fracture fixation, spinal fixation, and abdominal wall repair^4,10^. While commercially available polyester devices have seen a considerable amount of use in the biomedical field, their *in vivo* performance can be difficult to predict and evaluate due to the complex biological environment associated with tissues^10,11^. Therefore, the real-time monitoring of these devices is critical in order to understand their performance and fate in the body. The major drawback of polyester-based devices is that they lack inherent contrast imaging properties, making it difficult to visualize the area of interest. Imaging techniques are useful only when the intensity of a signal is sufficient enough to distinguish the target from surrounding tissues or materials. This issue becomes even more evident when imaging materials through deep tissue or when monitoring minor defects in biomaterials^12^. Recent advancements have addressed such problems through the improvement of contrast-enhancing agents, which can enrich the quality of images^4^. Iodine-containing small organic compounds remain at the forefront as contrast media due to the ability of heavy iodine atoms to highly absorb X-rays.

With this overall strategy in mind, our team has generated degradable iodine-bearing polyesters by utilizing conjugation strategies^13^ and oxime “Click” ligation reactions^14^. Most recently, we conducted a post-polymerization modification reaction between poly(ε-caprolactones) and iodinated hydroxylamines^15^. Herein, we report the design, synthesis, and applicability of a series of new iodine-containing polyesters as X-ray contrast imaging agents.

## Materials and Methods

### Materials

All reagents and chemicals were obtained from commercial sources and used without further purification unless stated otherwise. Water was purified on a Millipore Direct-Q S Water Purification System. Acetonitrile was dried by refluxing over phosphorous pentoxide (P_2_O_5_) and distilling under nitrogen prior to use. Lactic acid, synthesized monomers, and Na_2_SO_4_ were vacuum-dried overnight in the reaction vessel before use. All isolated products were purified by flash column chromatography using silica gel SDS 60 C.C. 40–63 μm. All known compounds and starting materials had ^1^H NMR and ^13^C NMR spectra consistent with previous literature reports. ^1^H and ^13^C NMR spectra were recorded at ambient temperature on a 500 MHz NMR spectrometer (Bruker). Proton and carbon chemical shifts were reported in parts per million (ppm) downfield from tetramethylsilane (TMS) with reference to the deuterated solvent as the internal standard (i.e., δ 7.26 ppm for ^1^H NMR, 77 ppm for ^13^C NMR in CDCl_3_). Data are presented as follows: chemical shift, integration, multiplicity (s = singlet, d = doublet, t = triplet, q = quartet, br = broad, m = multiplet), and coupling constants (*J*, in Hertz). Infrared (IR) spectra, reported in cm^−1^, were collected using a Shimadzu IRAffinity-1S Fourier transform spectrophotometer. Bands are characterized as strong (s), medium (m), weak (w), and broad (br). Melting points were recorded on a DigiMelt MPA160 apparatus.

A Tingle 325MVET X-ray machine was used to perform the X-ray imaging (51 kVp, 300 mA, and 5 millisecond exposure time). Due to its lack of radio-opaque properties and frequent use in biomedical applications, PLA was chosen as the control. All X-ray images were processed, and image intensities quantified using ImageJ (NIH) normalized to unmodified polymer.

### Synthesis

#### α-hydroxy-4-iodo-benzenepropionic acid (2); Typical Procedure

To a flame dried round bottom flask equipped with a stir bar was added 4-iodo-L-phenylalanine (17.2 mmol, 5.0 g, 1.0 equiv) and 0.5 M aq. H_2_SO_4_ (34.4 mmol, 2.0 equiv)^16^. The reaction was stirred for 10 min or until the solution was homogeneous. The solution was cooled to 0 °C and a solution of sodium nitrite (7.1 g, 103 mmol, 6.0 equiv) in H_2_O (50 mL) was added dropwise. The reaction was stirred at 0 °C for an additional 4 h, and then allowed to warm to room temperature. The resulting solution was stirred at room temperature for 24 h (monitored completion by TLC). The reaction mixture was extracted with diethyl ether (3 × 50 mL), and the organic phases were combined and washed with sat. aq. brine (50 mL) and dried over anhydrous sodium sulfate. The drying agent was filtered off, and the filtrate was concentrated *in vacuo*. The crude product was recrystallized from hexane-diethyl ether to give α-hydroxy-4-iodo-benzenepropionic acid as a yellowish white solid (74%, 3.7 g).

^1^H NMR (500 MHz, DMSO-d6): δ 2.75 (dd, 1 H, ^1^*J* = 13.92, ^2^*J* = 4.32 Hz), 2.93 (dd, 1 H, ^1^*J* = 13.7, ^2^*J* = 8.3 Hz), 4.16 (dd, 1 H, ^1^*J* = 7.9, ^2^*J* = 4.4 Hz),7.06 (d, 2 H, ^1^*J* = 7.8 Hz), 7.62 (d, 2 H, ^1^*J* = 7.8 Hz) ppm.

^13^C NMR (125 MHz, DMSO-d6): δ 64.9, 70.6, 91.9, 131.9, 136.7, 137.9, 174.9 ppm.

#### (3S)-3-(4-iodobenzyl)-6-methyl-1,4-dioxane-2,5-dione (3); Typical Procedure

To a flame dried round bottom flask equipped with a stir bar was added α-hydroxy-4-iodo-benzenepropionic acid (5.1 mmol, 1.5 g, 1.0 equiv), triethylamine (0.79 mL, 5.6 mmol, 1.1 equiv), and dry MeCN (25 mL) while under argon^17^. This solution was cooled to 0 °C, followed by the dropwise addition of 2-bromopropionyl chloride (0.57 mL, 5.6 mmol, 1.1 equiv). The mixture was stirred for 30 min. Triethylamine (0.79 mL, 5.6 mmol, 1.1 equiv) was added and the reaction was stirred at 70 °C for 3–5 h. The reaction was cooled to room temperature, quenched with 1 M aq. HCl (25 mL) and extracted with EtOAc (3 × 25 mL). The combined organic phases were washed with H_2_O (25 mL), sat. aq. brine (25 mL), and dried over anhydrous sodium sulfate. The drying agent was filtered off, and the filtrate was concentrated *in vacuo*. The crude product was purified by column chromatography (gradient, 100% hexanes to 70:30 hexanes:EtOAc) as a yellow oil, which solidified upon storage in the freezer (M.p. 121–122 °C, 58%, 1.03 g). Rf = 0.26 (70:30 hexanes:EtOAc)

^1^H NMR (500 MHz, CDCl_3_): δ 1.60 (d, 3 H, ^1^*J* = 6.7 Hz), 3.19 (dd, 1 H, ^1^*J* = 14.8, ^2^J = 7.5 Hz), 3.40 (dd, 1 H, ^1^*J* = 14.9, ^2^*J* = 4.0 Hz) 4.96 (q, 1 H ^1^*J* = 6.7 Hz), 5.06 (dd, 1 H, ^1^*J* = 3.9, ^2^*J* = 7.7 Hz), 7.08 (d, ^1^*J* = 8.3 Hz), 7.66 (d, ^1^*J* = 8.3 Hz) ppm.

^13^C NMR (125 MHz, CDCl_3_): δ 15.9, 35.7, 72.5, 76.1, 93.1, 131.9, 134.2, 137.8, 166.0, 166.7 ppm.

IR (neat): 2995 (w), 2932 (w), 1769 (s), 1738 (s), 1485 (w), 1250 (s) cm^−1^.

#### (S)-2-((R)-2-bromopropanamido)-3-(4-iodophenyl)propionic acid (4); Typical Procedure

To a flame dried round bottom flask equipped with a stir bar was added 4-iodo-L-phenylalanine (6.9 mmol, 2.0 g, 1.0 equiv), and 20 mL H_2_O:Et_2_O (1:1)^18–20^. To this solution was added 4 M aq. NaOH (8 mL) and stirred until all solids dissolved. This solution was then cooled to 0 °C. In a separate flame dried vial was added 2-bromopropionyl chloride (0.76 mL, 7.6 mmol, 1.1 equiv) and 4 M aq. NaOH (8 mL). The solution in the vial was added dropwise to the round bottom flask while maintaining a temperature of 0 °C and a pH of 11 throughout the addition (*i.e*. 4 M aq. NaOH was added via dropping funnel to maintain pH). After completion of the reaction (monitored by TLC), the reaction was warmed to room temperature and the ether layer was separated in a separatory funnel. The aqueous layer was acidified with concentrated HCl to pH of 1, and extracted with ethyl acetate (4 × 25 mL). The combined organic phases were washed with sat. aq. brine and dried over anhydrous sodium sulfate. The drying agent was filtered off, and the filtrate was concentrated *in vacuo*. The white, solid crude product was placed under high vacuum and used in next step without further purification.

#### (3S,6S)-3-(4-iodobenzyl)-6-methylmorpholine-2,5-dione (5); Typical Procedure

To a flame dried round bottom flask equipped with a stir bar was added crude (S)-2-((R)-2-bromopropanamido)-3-(4-iodophenyl)propionic acid (6.8 mmol, 2.9 g, 1 equiv) and DMF (25–30 mL) followed by triethylamine (1.04 mL, 7.5 mmol, 1.1 equiv)^18–21^. The mixture was heated to 90 °C for 12 h under N_2_. When the reaction was complete (as monitored by TLC), the mixture was allowed to stand overnight in the freezer. The crystallized salt and DMF/TEA were filtered, and the filtrate was concentrated *in vacuo*. Residual DMF was removed by adding toluene to the sample, followed by rotary evaporation (repeated 4 times). Further purification was performed by recrystallizing the crude isolate from chloroform and cold Et_2_O to furnish a white solid (M.p. 147–148 °C, 1.12 g, 48% over 2 steps).

^1^H NMR (500 MHz, DMSO-d6): δ 1.13 (d, 3 H, ^1^*J* = 6.9 Hz), 3.02 (d, 2 H, ^1^*J* = 5.1 Hz), 4.67 (t, 1 H, ^1^*J* = 5.0 Hz), 4.99 (q, 1 H, ^1^*J* = 6.8 Hz), 7.09 (d, 2 H, ^1^*J* = 8.1 Hz), 7.68 (d, 2 H, ^1^*J* = 8.2 Hz) ppm.

^13^C NMR (125 MHz, CDCl_3_): δ 16.8, 36.1, 54.2, 74.6, 93.3, 132.7, 136.5, 137.4, 168.5, 168.6 ppm.

IR (neat): 3202 (br), 1747 (s), 1694 (s), 1483 (w), 1375 (m), 1315 (m) cm^−1^.

#### (S)-2-(2-chloroacetamido)-3-(4-iodophenyl)propionic acid (6)

Following a procedure analogous to the preparation of **4**, **6** was obtained by using chloroacetyl chloride in place of 2-bromopropionyl chloride^18–21^. The yellow colored crude product was used in the next step without further purification.

#### (S)-3-(4-iodobenzyl)morpholine-2,5-dione (7)

Following a procedure analogous to the preparation of **5**, but using **6** as the starting material, the product was obtained as an off-white solid^18–21^. (M.p. 156–157 °C, 1.12 g, 41% over 2 steps).

^1^H NMR (500 MHz, CDCl_3_): δ 3.17–3.19 (dd, 2 H, ^1^J = 2.32 Hz), 4.00–4.06 (d, 1 H, ^1^J = 16.54 Hz), 4.46–4.50 (dd, 1 H, ^1^J = 3.60 Hz), 4.52–4.58 (d, 1 H, ^1^J = 16.54 Hz), 6.99–7.01 (d, 2 H, ^1^J = 8.19 Hz), 7.11 (br, 1 H), 7.69–7.72 (d, 2 H, ^1^J = 8.16 Hz) ppm.

^13^C NMR (125 MHz, CDCl_3_): δ 39.2, 54.4, 67.0, 93.8, 131.6, 133.7, 138.4, 165.8, 165.9 ppm.

IR (neat): 3205 (br), 2922 (m), 1755 (s), 1678 (s), 1485 (m), 1337 (m) cm^−1^.

#### 1-Cyclohexenylpyrrolidine (9); Typical Procedure

While under N_2_, a flame dried round bottom flask equipped with a stir bar and Dean-Stark trap (with activated 4 Å molecular sieves) was charged with cyclohexanone (4.22 mL, 40.8 mmol, 1 equiv), dry toluene (20 mL), and pyrrolidine (6.02 mL, 73.4 mmol, 1.8 equiv)^22^. The mixture was refluxed under N_2_ for 5–7 h. The solvent and excess pyrrolidine were removed *in vacuo*. The resulting crude, colorless oil (5.8 g, 94%) was used without further purification in the subsequent transformation.

^1^H NMR (500 MHz, CDCl_3_): δ 1.38–1.43 (m, 2 H), 1.52–1.55 (m, 2 H), 1.66–1.68 (m, 4 H), 1.95–1.96 (m, 2 H), 2.02–2.03 (m, 2 H), 2.84–2.85 (m, 4 H), 4.10–4.16 (m, 1 H) ppm.

^13^C NMR (125 MHz, CDCl_3_): δ 22.9, 23.3, 24.5, 26.9, 27.4, 47.2, 93.3, 142.9 ppm.

#### 2-(4-iodobenzyl)cyclohexan-1-one (10); Typical procedure

While under N_2_, a flame dried round bottom flask equipped with a stir bar was charged with 4-iodobenzyl bromide (4.9 g, 16.5 mmol, 1 equiv) and dry toluene (30 mL)^23^. After dissolution, the flask was charged with 1-cyclohexenylpyrrolidine **9** (2.5 g, 16.5 mmol, 1 equiv). The mixture was refluxed under N_2_ for 18 h. H_2_O (30 mL) was added and the solution was heated for an additional hour. The solvent was evaporated under vacuum, and the residue was extracted with diethyl ether. The ether phase was washed consecutively with 5% aq. HCl, 5% aq. NaHCO_3_ solution, and water, then dried, and evaporated. The residue was purified via silica gel chromatography eluted with hexanes/ethyl acetate (9:1) to provide a white solid (3.7 g, 71%).

^1^H NMR (500 MHz, CDCl_3_): δ 1.15–3.02 (m, 11 H), 6.79 (d, 2 H, ^1^*J* = 8.3 Hz), 7.42 (d, 2 H, ^1^*J* = 8.3 Hz) ppm.

^13^C NMR (125 MHz, CDCl_3_): δ 25.1, 27.9, 33.5, 35.1, 42.1, 52.0, 91.2, 131.4, 137.2, 140.1, 211.4 ppm.

#### 7-(4-iodobenzyl)oxepan-2-one (11); Typical procedure

While under N_2_, a flame dried round bottom flask equipped with a stir bar was charged with 2-(4-iodobenzyl)cyclohexan-1-one (1.5 g, 4.8 mmol, 1 equiv) and CHCl_3_ (50 mL)^24,25^. The solution was cooled to 0 °C, and to this solution was added *m*CPBA (77% reagent) (14.3 mmol, 3 equiv). The mixture was stirred at room temperature for 2–3 d (monitored by TLC), and the reaction mixture was quenched with a sat. aq. Na_2_S_2_O_3_ solution. The aqueous layer was extracted with 3X CHCl_3_. The combined organic layer was washed with sat. aq. NaHCO_3_ and sat. aq. brine. The organic layers were dried over Na_2_SO_4_, filtered, and concentrated *in vacuo*. The residue was purified via silica gel chromatography eluted with a gradient of hexanes to hexanes:ethyl acetate (2:8) to provide a colorless oil (1.07 g, 68%). Rf = 0.69 (1:9 hexanes:EtOAc)

^1^H NMR (500 MHz, CDCl_3_): δ 1.33–2.78 (m, 10 H), 4.28–4.3 (m, 1 H), 6.85 (d, 2 H, ^1^*J* = 8.1 Hz), 7.40 (d, 2 H, ^1^*J* = 8.2 Hz) ppm.

^13^C NMR (125 MHz, CDCl3): δ 22.9, 28.3, 33.8, 34.9, 42.1, 80.7, 92.2, 131.7, 136.9, 137.6, 175.2 ppm.

IR (neat): 2924 (w), 2857 (w), 1726 (s), 1483 (w), 1173 (m), 1007 (w) cm^−1^.

### Example Procedure for Ring-Opening Polymerization

Polymers were synthesized using ring opening polymerization with lactic acid as the initiator and tin (II) 2-ethylhexanoate as the catalyst. Prior to use, the monomer, lactic acid, sodium sulfate, and stir bar were vacuum-dried overnight in the reaction vessel. The reaction vessel was equipped with a reflux condenser, and the reagents were dissolved in anhydrous toluene while under a nitrogen atmosphere. When the reaction reached 120 °C, tin(II) 2-ethylhexanoate was added, and the reaction was allowed to stir at this temperature for 24 h. The resulting polymer was partitioned between chloroform and water, and the aqueous phase was extracted three times with chloroform. The chloroform phases were combined and dried over MgSO_4_. The filtrate was collected and the desired polymer was precipitated from cold methanol.

### X-ray imaging

X-ray imaging was performed using a Tingle 325MVET X-ray machine with 51 kVp, 300 mA and 5 ms exposure time. 2.6.

### *In vitro* polymer degradation analysis with X-ray imaging

iPLA was fabricated into pellets (25 mg) by heating and molding the polyester into the desired shape. Pellets were placed into plastic tubes filled with PBS (pH = 7.4), and then incubated at 37 °C and 5% CO_2_. After being placed in PBS and each day following, the plates were imaged using X-ray. Each day, the PBS was replaced. The controls for this study were discs made from PDLLA, which is known to have no imaging properties. Three repeats were used. Statistical analyses were performed using a two-tailed t-test and statistical significance was set at p < 0.05. Statistical significance is denoted by ‘*’.

### *In vitro* X-ray imaging of polymeric discs through tissue

iPLA was fabricated into dry powder. Chicken tissue was sectioned into slices of known thickness 2 cm and 5 cm). The chicken sections were placed on top of the iPLA powder to simulate increases in tissue depth inside the human body and imaged using X-ray.

## Results and Discussion

**Figure 1:**
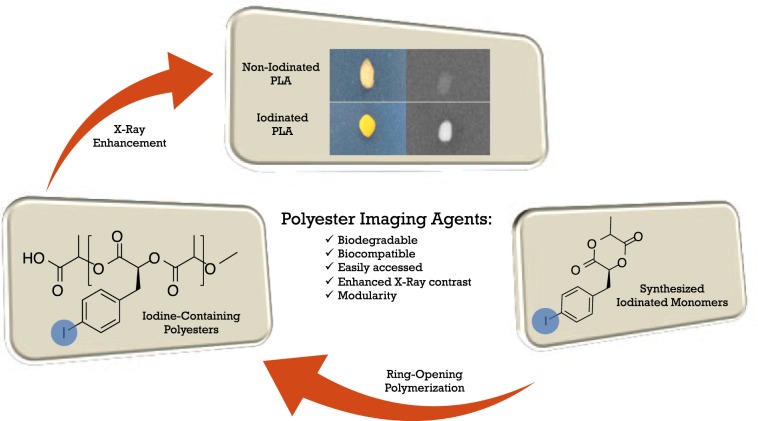
Synthesis of iodinated lactide monomers bearing a 4-iodobenzyl moiety to impart X-ray opacity of the resulting polymer materials with X-ray contrast properties.

We designed four target monomers each bearing a 4-iodobenzyl moiety to impart X-ray opacity of the resulting polymer materials (Fig. 1).

**Figure 2:**
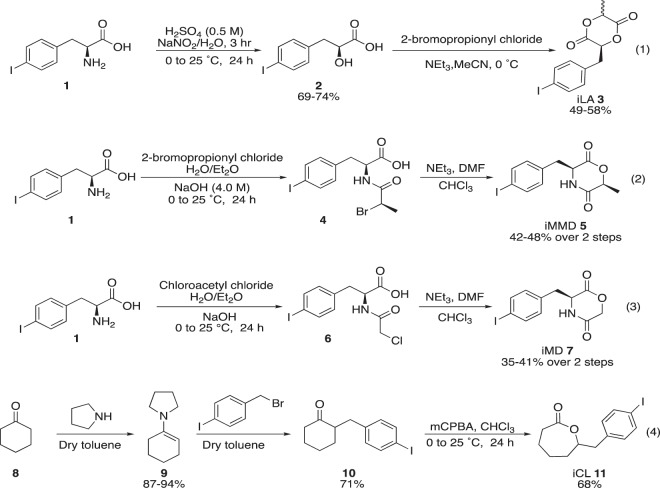
Synthesis of iodinated monomers.

Specifically, we designed lactide **3**, morpholinediones **5** and **7**, and caprolactone **11** (Fig. 2). These monomers were designed with the twin goals of maintaining high reactivity in the tin-catalyzed polymerization reaction, but also incorporated the aryl-iodo motif by the formation of a stable, non-reversible carbon-carbon bond. The syntheses of lactide **3** and morpholinediones **5** and **7** all commenced with commercially available 4-iodophenylalanine (**1**). Lactide **3** arose in two steps. Diazotization/hydrolysis of **1** to provide the α-hydroxy carboxylic acid **2** was followed by acylation/substitution with 2-bromopropionyl chloride, thus resulting in 4-iodo-benzyl lactide **3** (34–43% over two steps). The methyl substituted morpholinedione **5** (42–48% over two steps) and non-substituted derivative **7** (35–41% over two steps) were synthesized through an acylation/base-induced cyclization sequence employing 2-bromopropionyl chloride and chloroacetyl chloride, respectively. Lastly, the 4-iodo-benzyl-caprolactone **11** was assembled in three straightforward steps. First, pyrrolidine and cyclohexanone were refluxed in toluene to afford enamine **9** in 87–94% yield. Second, the aryl-iodo moiety was incorporated via enamine alkylation with 4-iodo-benzylbromide to provide substituted ketone **10** in 71% yield. The final step supplied the target 4-iodobenzyl caprolactone **11** in 68% yield by means of a Baeyer-Villiger oxidation with *m-*chloroperbenzoic acid.

**Figure 3:**
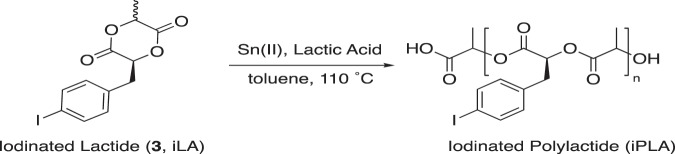
Tin-mediated ring opening polymerization.

The corresponding polyesters were synthesized using standard ring-opening polymerization techniques (Fig. 3). To target a predictable ratio of copolymers, a conventional thermal ring-opening polymerization with tin (II) 2-ethylhexanoate was exploited^4,26^. Isolation of the final products was carried out by means of precipitation in cold methanol followed by centrifugation at –9 °C for 5 min followed by freeze-drying for 3 days. The polymers were then stored at –20 °C before use.

**Figure 4:**
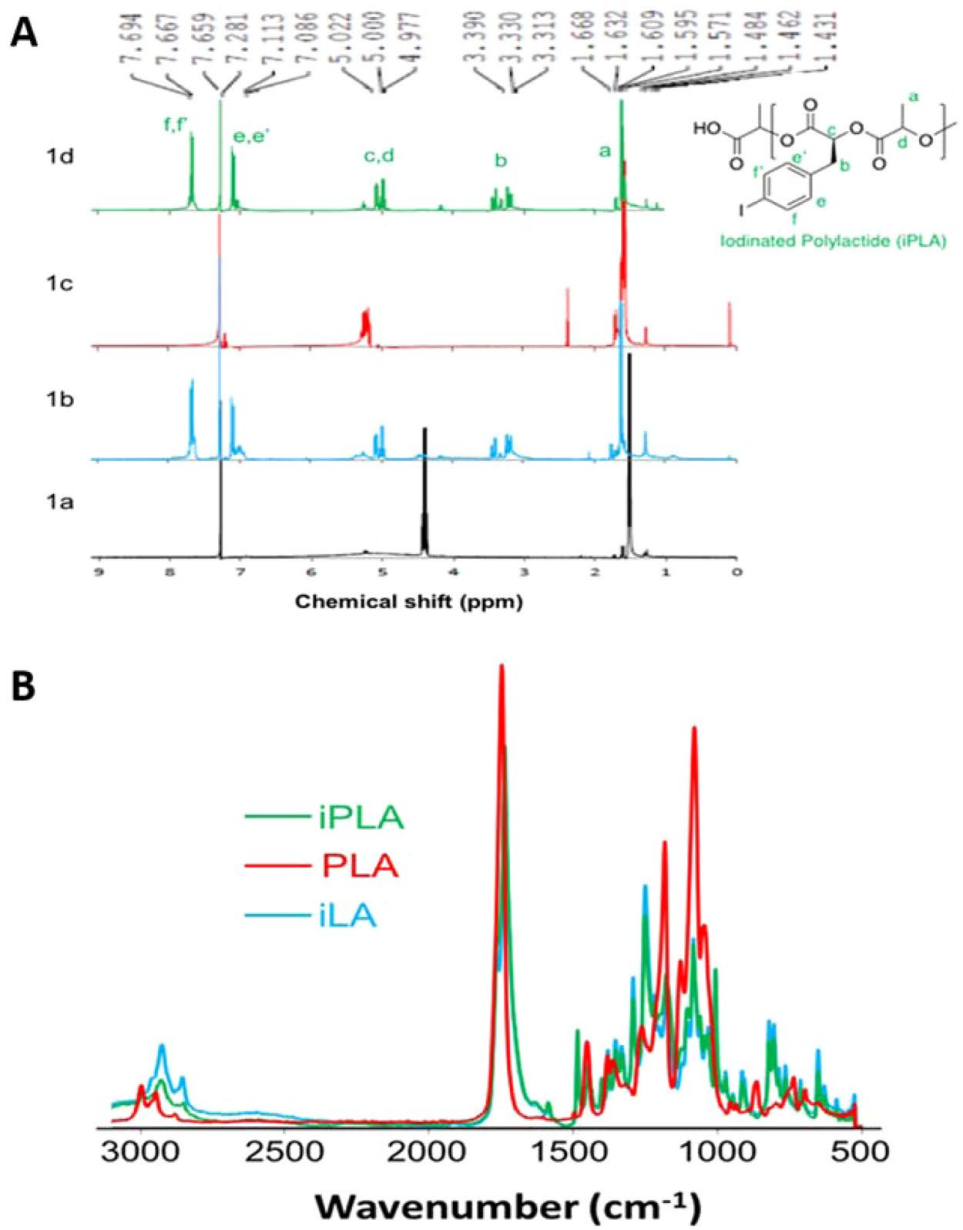
(**A**) ^1^H NMR spectral overlay of (1a) lactide, (1b) aryl-iodo lactide (iLA), (1c) poly(lactic) acid (PLA), and (1d) aryl-iodo poly(lactic) acid (iPLA). (**B**) FTIR characterization of the aryl-iodo lactide (iLA), poly(lactic) acid (PLA), and the aryl-iodo poly(lactic) acid (iPLA).

The resulting iodinated polyesters were characterized by ^1^H NMR and FTIR spectroscopy. An illustrative example of the ^1^H NMR and FTIR spectra comparing the iodinated poly(lactic) acid and poly(lactic) acid as a control, along with its monomeric precursor **3**, can be seen in Fig. 4.

The presence of the 4-iodobenzyl moiety in monomeric aryl-iodo lactide **3** is readily apparent based on the downfield resonances at 7.2 and 7.7 ppm, which are still present in the polymerized (iPLA) ^1^H NMR spectrum, indicating that the aryl-iodo functionality is stable throughout the ring-opening process. Additionally, the downfield shift of the alpha proton signal in the PLA spectra confirm successful polymerization (Fig. 4A**)**.

Furthermore, the FTIR spectra displays a peak at 1755 cm^−1^ that corresponds to the carbonyl stretching frequency (Fig. 4B). A peak at 2955 cm^−1^, corresponding to sp^2^ C-H stretches, is apparent in both iLA and iPLA, and absent in the case of PLA.

**Figure 5:**
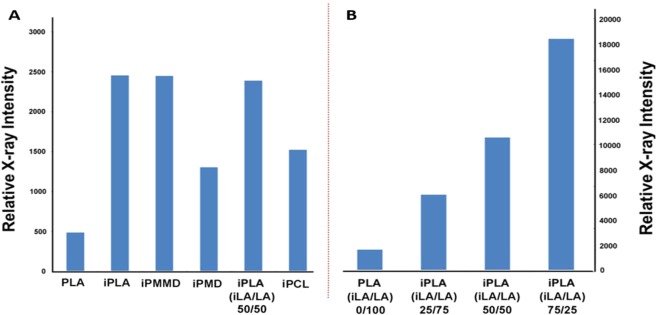
(**A**) Relative X-ray intensity of iodinated polymers compared to PLA. (**B**) Relative X-ray intensity of iodinated PLA and PLA copolymer with varying ratios.

Next, the X-ray contrast properties of the synthesized iodinated polyesters were evaluated using X-ray imaging methodology (recorded in Hounsfield units). Figure 5 illustrates that the aryl-iodo containing polymers have an increased relative X-ray intensity when compared to unmodified iodine-free PLA (Fig. 5A). The significance of substituents on the polymeric backbone can be seen by comparing the substituted and non-substituted morpholinedione polyesters. The polymer of the iodine-containing modified morpholinedione (iPMMD), which bears an additional methyl-substituent, contains a lower iodine-weight concentration than the non-substituted iodinated polymer of morpholinedione (iPMD), and in theory should have a lower X-ray intensity. However, iPMMD is able to absorb X-rays more efficiently than the non-methylated analog (iPMD). The iodinated lactide and substituted morpholinedione polyesters exhibited comparable X-ray intensities, which can be attributed to their similar chemical structures and molecular weights.

The 4-iodobenzylcaprolactone (iCL) monomer, provided an iodine-containing poly(-ε-caprolactone) (iPCL). Unlike commonly used PLA/PLG polymers, PCL polymers slowly degrade into less acidic, low molecular weight by-products, preventing the generation of a toxic environment^27^. Although the relative X-ray intensity of iPCL was lower than the iPLA and iPMMD materials, the access to a class of bioresorbable X-ray enhancing polycaprolactones may be useful for some medical applications^4^. We selected the iPLA material for further studies based on its strong X-ray contrasting properties coupled with its relative ease of synthesis from economical, readily available starting materials.

A poly(lactic acid) copolymer iPLA(iLA/LA) which incorporated a 50:50 ratio of lactic acid to iodinated lactic acid was generated, and was shown to have a comparable relative X-ray intensity (~2445 HU) to the fully iodinated PLA (~2450 HU). We were motivated by the fact that the copolymer could achieve similar X-ray intensity as the homopolymer iPLA, yet it bears only half the amount of iodine possibly due to higher reactivity with LA monomers resulting in a copolymer with a larger molecular weight than the iPLA homopolymer. To further investigate the strategy of incorporating non-iodinated subunits into contrast enhancing polymers, we copolymerized lactide with varying ratios of the iodinated monomer (Fig. 5B).

As expected, there was a direct correlation between the X-ray intensity and the amount of iodinated monomer used. The resulting intensity values in Fig. 5B are consistent with a gradual increase of iLA monomer in the copolymerization process of poly(lactic) acid. PLA (100% LA), PLA (iLA: LA; 25:75), PLA (iLA: LA; 50:50), PLA (iLA: LA; 75:25) showed ~1800 HU, ~6000 HU, ~10700, ~18550 HU respectively.

**Figure 6:**
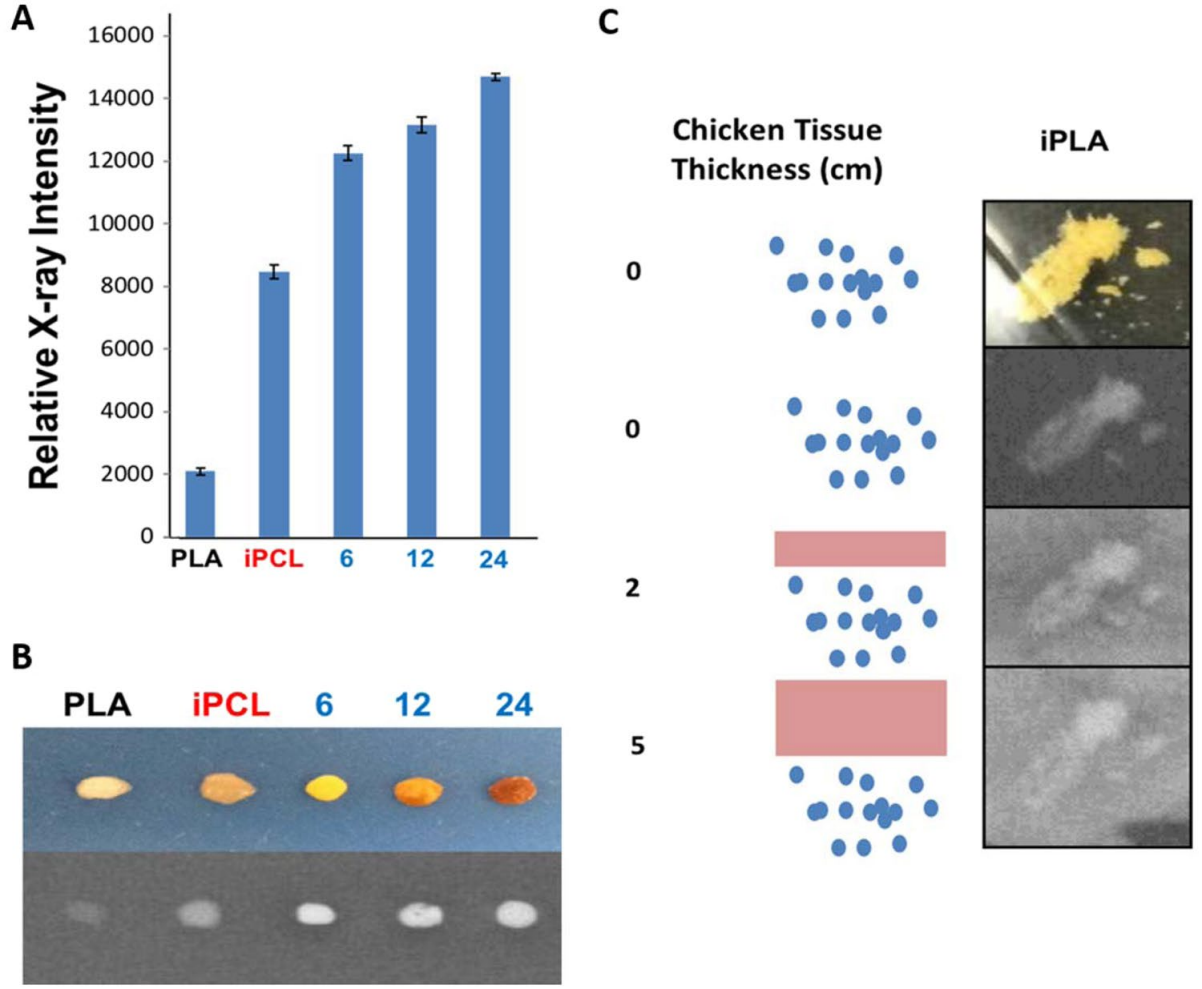
Quantitative measurements of relative X-ray intensity. (**A**) Relative X-ray intensity of iodinated poly(lactic) acid (iPLA) synthesized using various reaction times, in comparison to iodinated polycaprolactone (iPCL). Three repeats were used. Statistical analyses were performed using a two-tailed t-test and statistical significance was set at p < 0.05. (**B**) Depiction of polymeric pellets (10 mg) composed of poly(lactic) acid (PLA), iodinated polycaprolactone (iPCL), and iodinated poly(lactic) acid (iPLA) copolymers synthesized using 6, 12, and 24 hours reaction times. (C) *In vitro* imaging of iPLA powder through different depth of chicken tissue (0 cm, 2 cm, and 5 cm thick chicken tissue).

To validate the successful incorporation of iLA into the copolymer, we probed the relative X-ray intensity of the copolymerization at 6, 12, and 24-hour time points (Fig. 6A).

The increase in X-ray intensity over time confirms the effective incorporation of iLA into the resulting copolymer. Figure 6B depicts the generated polymeric pellets used in the copolymerization time study, taking the PLA with low contrast as a control showing the gradual increase in the intensity over reaction time.

A persistent obstacle frequently encountered in medical imaging is the visualization of biomedical devices through deep tissue. In order to explore the potential of the synthesized polymeric materials to help overcome this obstacle, *in vitro* imaging of iPLA powder was performed through varying thicknesses (*i.e*. 0, 2, and 5 cm) of chicken tissue. As seen in Fig. 6C, effective visualization of the iPLA material was observed through 5 cm-thick chicken tissue, confirming the potential of the polymeric material for deep tissue imaging applications.

**Figure 7:**
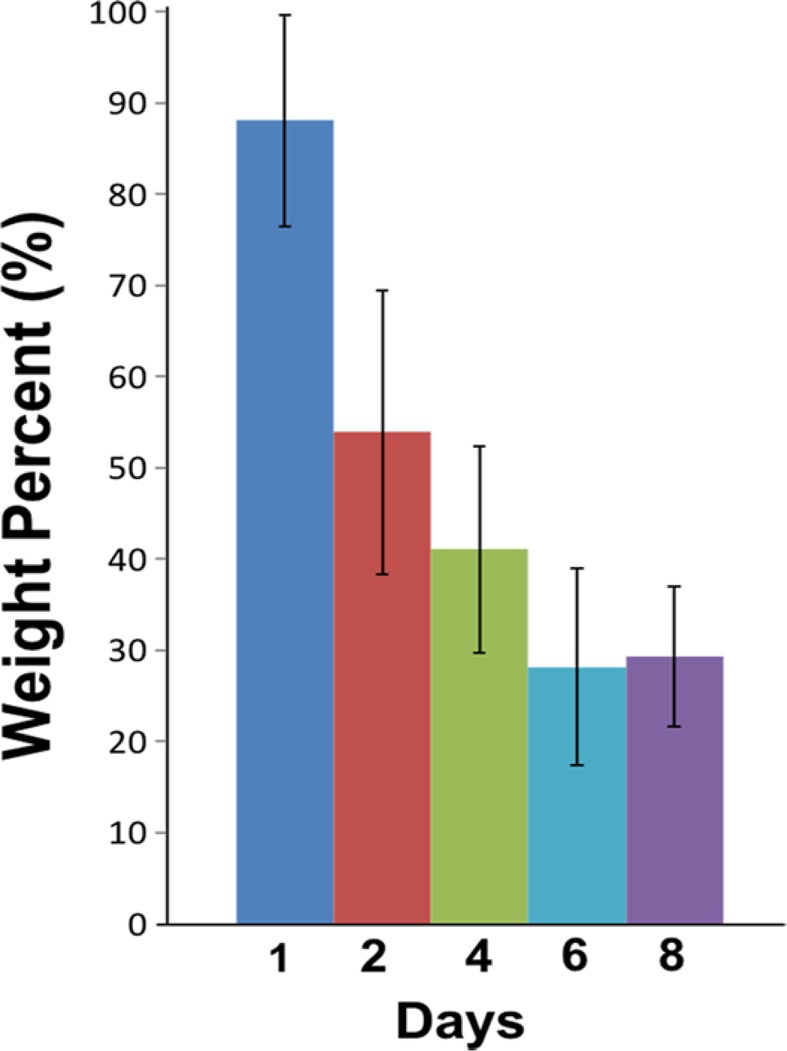
Degradation of iPLA pellets (25 mg) over time (days) which was incubated into PBS (pH 7.4) at 37 ^o^C.

Lastly, the weight loss of iPLA was monitored over an eight-day period, giving a degradation profile as seen in Fig. 7.

The overall decrease of weight percentage confirms that the aryl-iodo functionality does not disrupt the biodegradability of the polymer, and the hydrolysis of ester bonds release water soluble monomers and oligomers^28^. As described in Fig. 7, the degradation rate profile of iPLA gradually decreases with time until nearly 70% of its weight is lost after eight days.

## Conclusion

A series of monomers bearing a 4-iodoobenzyl moiety (i.e. lactides, morpholines and caprolactones) were synthesized leveraging straightforward transformations. A tin-mediated ring opening polymerization was then conducted, and the resulting polymers were characterized through nuclear magnetic resonance (NMR) and Fourier-transform infrared (FTIR) spectroscopy. The presented synthetic protocol is attractive because, unlike post-polymerization functionalization methodology of biomaterials such as polyesters, it incorporates the critical iodine atom into each monomeric unit, therefore maximizing iodine content within the resulting polymer.

The modified polyesters were evaluated as viable contrast X-ray imaging agents, with emphasis on their potential use in deep tissue imaging. The iodine-containing polyesters that exhibited the highest X-ray intensities were the modified iodo-morpholinedione polymer (iPMMD) and the iodo-poly(lactic) acid (iPLA). Evidently, the iPMMD can attain nearly double the X-ray intensity, in comparison to the non-substituted iodo-morpholinedione polymer (iPMD), when incorporating a methyl substituent opposite the iodobenzyl moiety. The copolymerization of the iodinated lactide and unmodified lactide (50:50) resulted in a polymer with significant X-ray intensity, and therefore demonstrates the possibility to assemble polyester copolymers with a combination of iodinated and unmodified monomers. The easily synthesized iPLA contrast agent was readily visualized through 5 cm of chicken tissue, validating its potential for deep tissue imaging applications. Lastly, we probed the degradation profile of iPLA, and confirmed that the covalently bound iodine does not perturb the biodegradability of the polyester. However, the rapid loss of X-Ray contrast properties over 1–2 weeks suggests that the molecular weight of the iodinated polyesters are expected to be small and suitable for rapid degradation applications including biodegradable nanoparticles X-ray contrast agent, biodegradable sutures, etc. Crosslinking or conjugation approaches could be used to increase the molecular weight for long term monitoring of biodegradable implants etc.

Iodine containing X-ray contrast agents have demonstrated clinical relevance within the field of medical imaging. Iodinated polymeric contrast-enhancing materials, being biodegradable, and therefore less toxic, have the potential to further improve medical imaging capabilities. This work demonstrates a highly concise and easily modified strategy to synthesize biodegradable and biocompatible X-ray visible materials.

## supplementary information

supplementary information
