# Supplementary material for: Iodinated Polyesters with Enhanced X-ray Contrast Properties for Biomedical Imaging
Source: Sci Rep. 2020 Jan 30;10:1508. doi: 10.1038/s41598-020-57720-5 (PMC6992777; doi:10.1038/s41598-020-57720-5)
Supplement: Supplementary file 1 — supplementary information [file 41598_2020_57720_MOESM1_ESM.pdf]

Electronic Supplementary Information

Iodinated Polyesters with Enhanced X-ray Contrast Properties for Biomedical Imaging

Timothy R. Lex, Beau R. Brummel, Mohamed F. Attia, Brooke A. Van Horn, Daniel. C. Whitehead,* Frank Alexis*

Email: falexis@yachaytech.edu.ec

E-mail: dwhiteh@clemson.edu

Table of Contents:

^{1}H and ^{13}C NMR spectra for **2**……………………………………………………………………S2

^{1}H and ^{13}C NMR spectra for **3**……………………………………………………………………S3

^{1}H and ^{13}C NMR spectra for **5**……………………………………………………………………S4

^{1}H and ^{13}C NMR spectra for **7**……………………………………………………………………S5

^{1}H and ^{13}C NMR spectra for **9**……………………………………………………………………S6

^{1}H and ^{13}C NMR spectra for **10**…………………………………………………………………S7

^{1}H and ^{13}C NMR spectra for **11**…………………………………………………………………S8

TL-1-103
DMSO

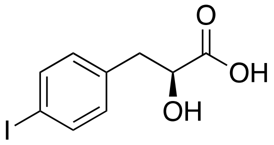

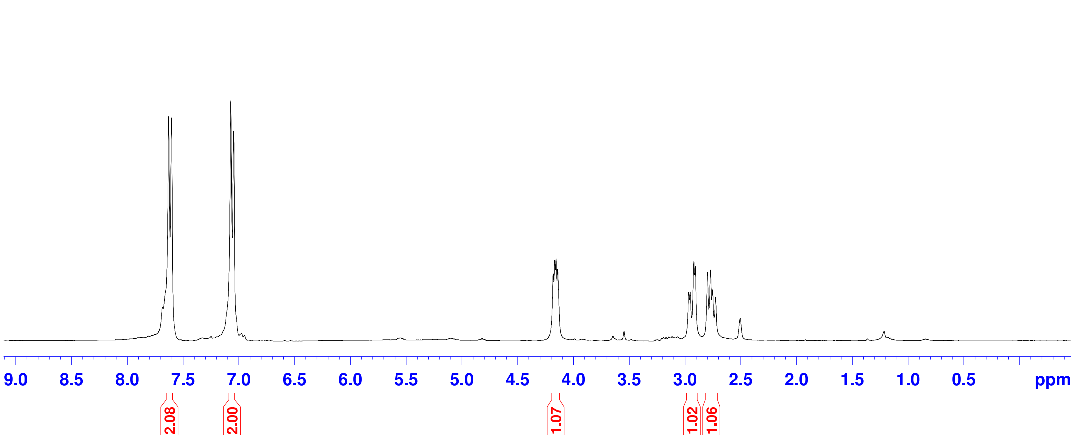

TL-I-103
DMSO

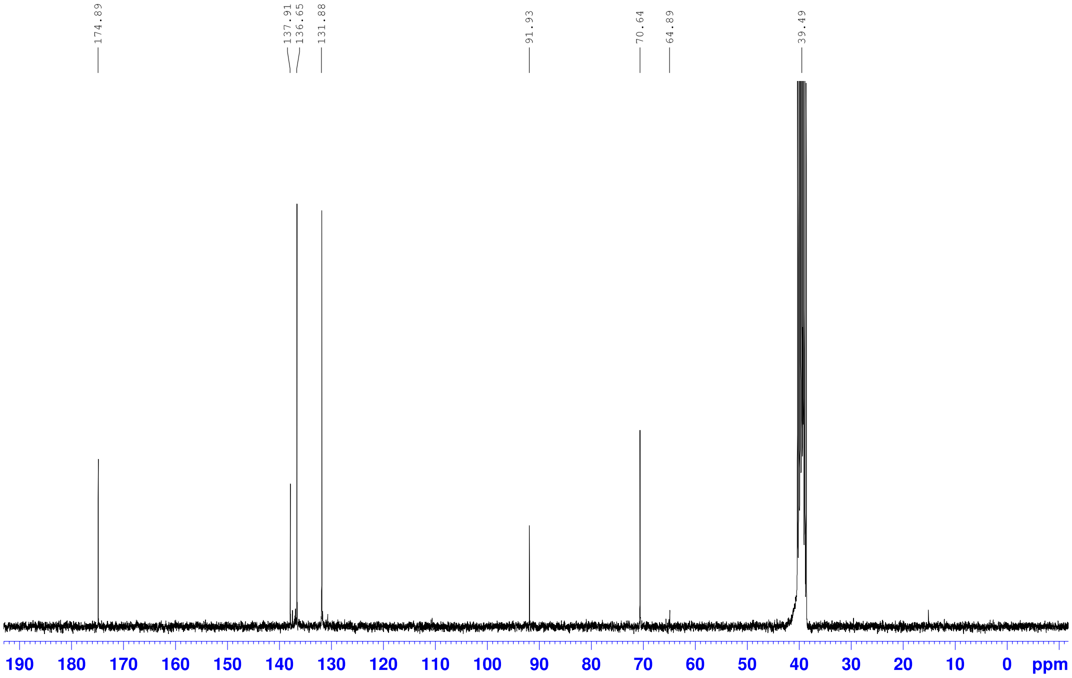

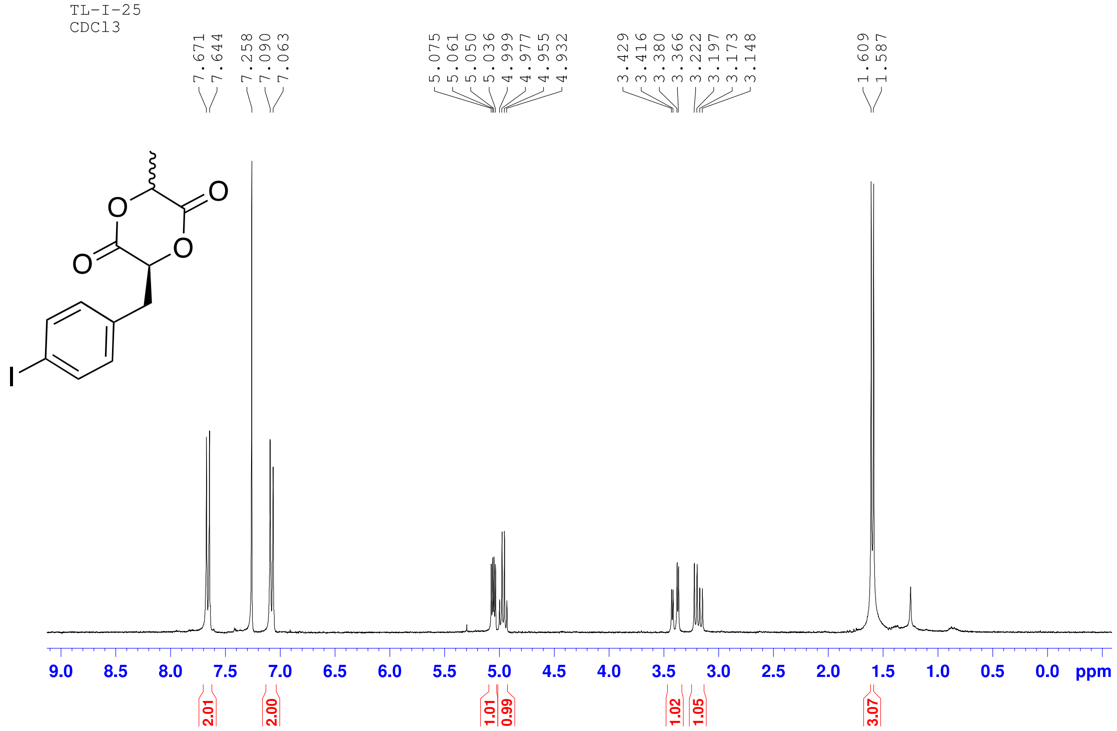
TL-I-25
CDCl3
7.671
7.644
7.258
7.090
7.063
5.075
5.061
5.050
5.036
4.999
4.977
4.955
4.932
3.429
3.416
3.380
3.366
3.222
3.197
3.173
3.148
1.609
1.587
O
O
O
O
I
9.0
8.5
8.0
7.5
7.0
6.5
6.0
5.5
5.0
4.5
4.0
3.5
3.0
2.5
2.0
1.5
1.0
0.5
0.0
ppm
2.01
2.00
1.01
0.99
1.02
1.05
3.07

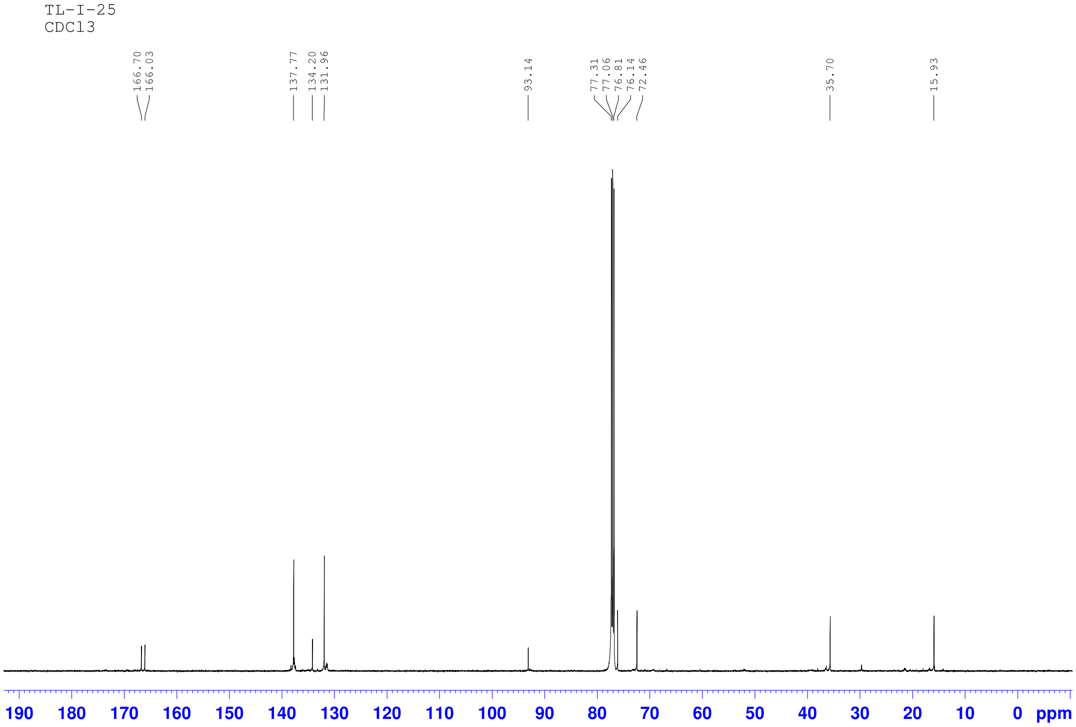
TL-I-25
CDCl3
166.70
166.03
137.77
134.20
131.96
93.14
77.31
77.06
76.81
76.14
72.46
35.70
15.93
190
180
170
160
150
140
130
120
110
100
90
80
70
60
50
40
30
20
10
0
ppm

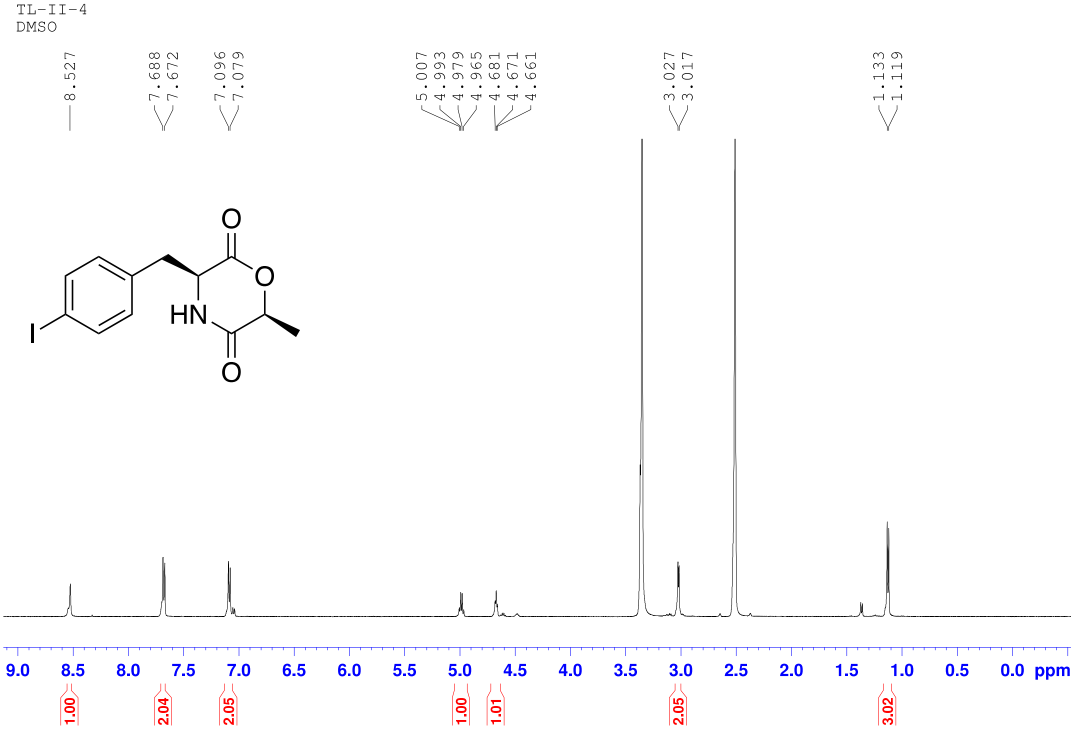
TL-II-4
DMSO
8.527
7.688
7.672
7.096
7.079
5.007
4.993
4.979
4.965
4.681
4.671
4.661
3.027
3.017
1.133
1.119
HN
O
9.0 8.5 8.0 7.5 7.0 6.5 6.0 5.5 5.0 4.5 4.0 3.5 3.0 2.5 2.0 1.5 1.0 0.5 0.0 ppm
1.00
2.04
2.05
1.00
1.01
2.05
3.02

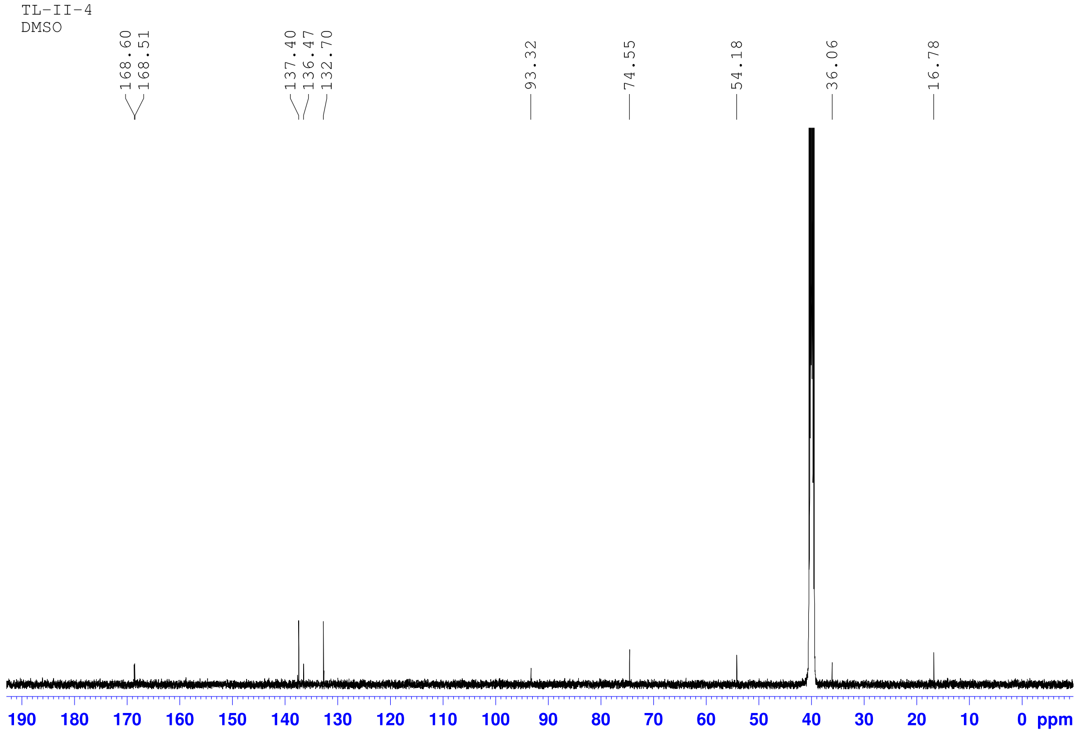
TL-II-4
DMSO
168.60
168.51
137.40
136.47
132.70
93.32
74.55
54.18
36.06
16.78
190 180 170 160 150 140 130 120 110 100 90 80 70 60 50 40 30 20 10 0 ppm

TL-V-107

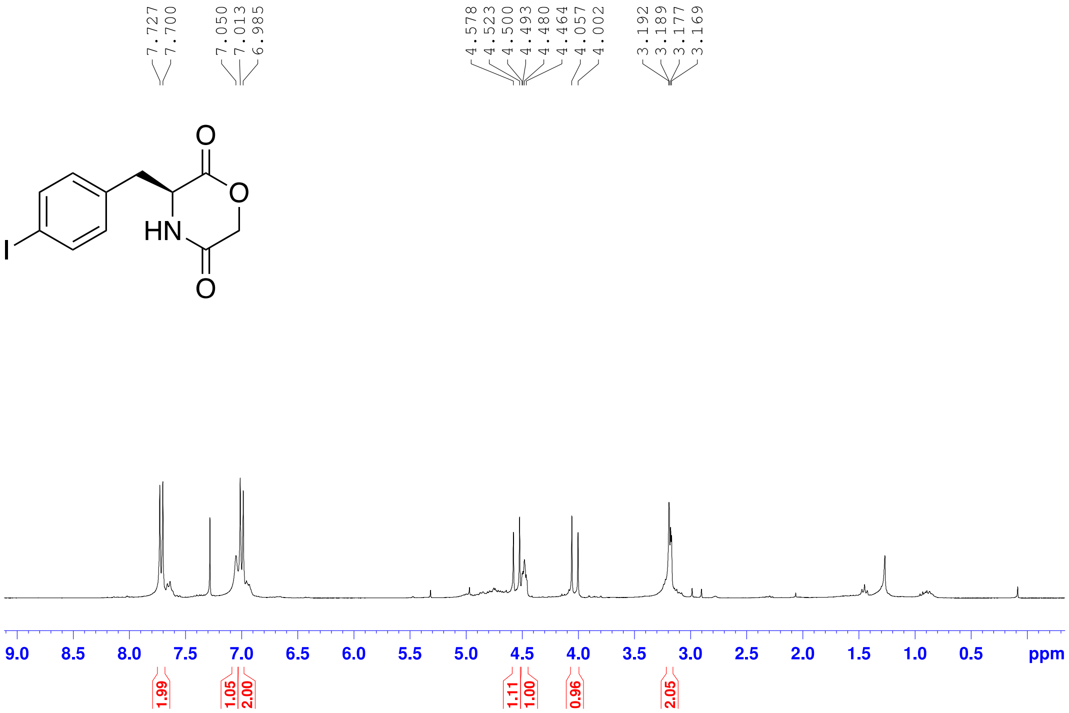

TL-V-107

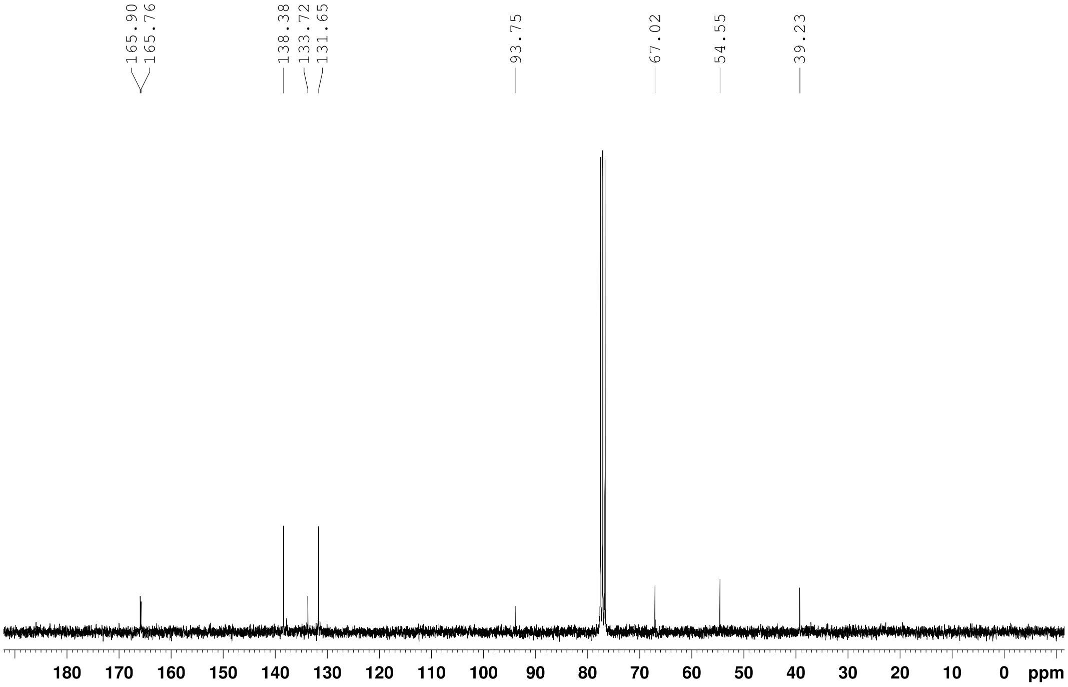

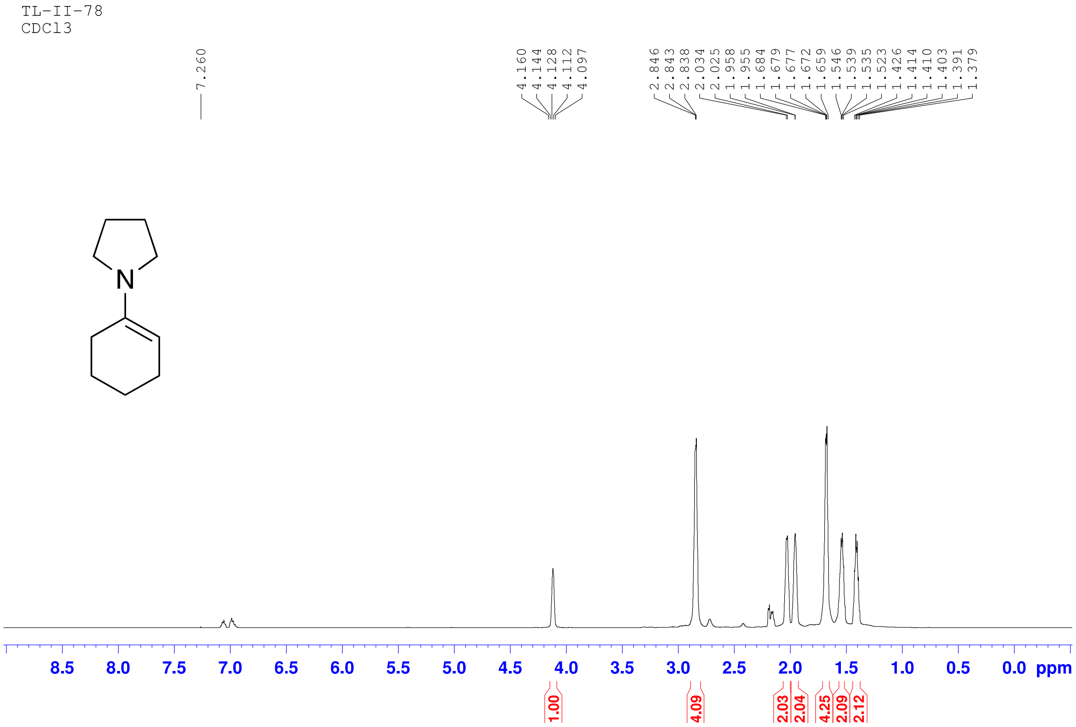
TL-II-78
CDCl3
7.260
4.160 4.144 4.128 4.112 4.097
2.846 2.843 2.838 2.034 2.025 1.958 1.955 1.684 1.679 1.677 1.672 1.659 1.546 1.539 1.535 1.523 1.426 1.414 1.410 1.403 1.391 1.379
N
8.5 8.0 7.5 7.0 6.5 6.0 5.5 5.0 4.5 4.0 3.5 3.0 2.5 2.0 1.5 1.0 0.5 0.0 ppm
1.00
4.09
2.03 2.04 4.25 2.09 2.12

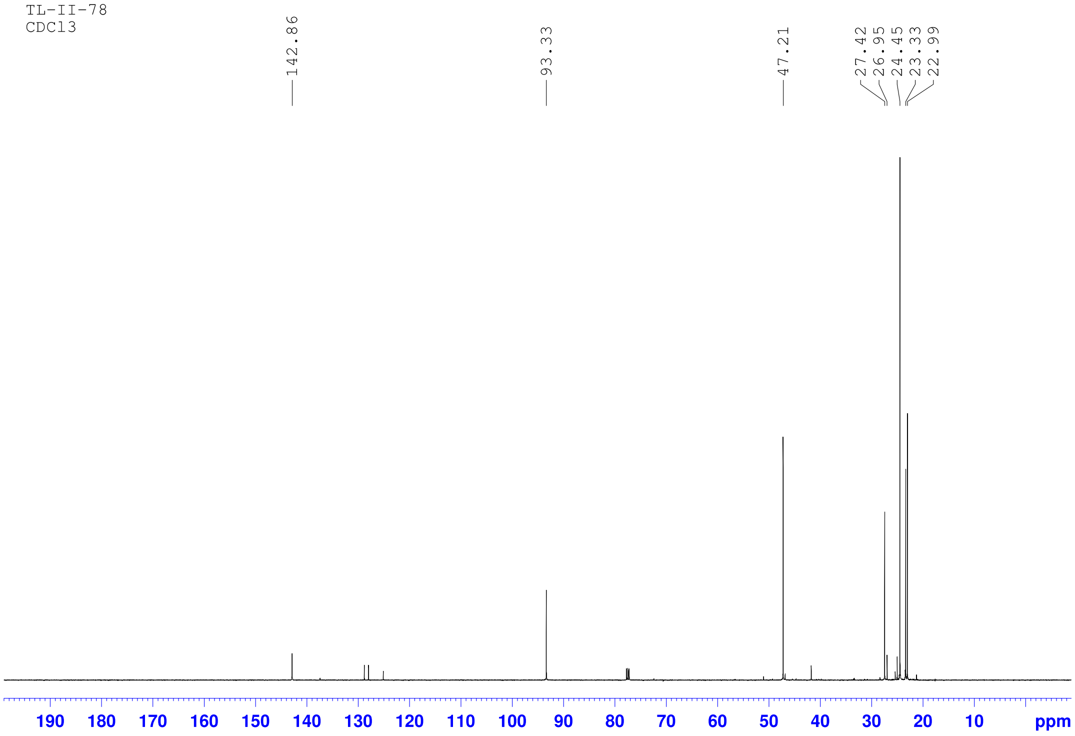
TL-II-78
CDCl3
142.86
93.33
47.21
27.42 26.95 24.45 23.33 22.99
190 180 170 160 150 140 130 120 110 100 90 80 70 60 50 40 30 20 10 ppm

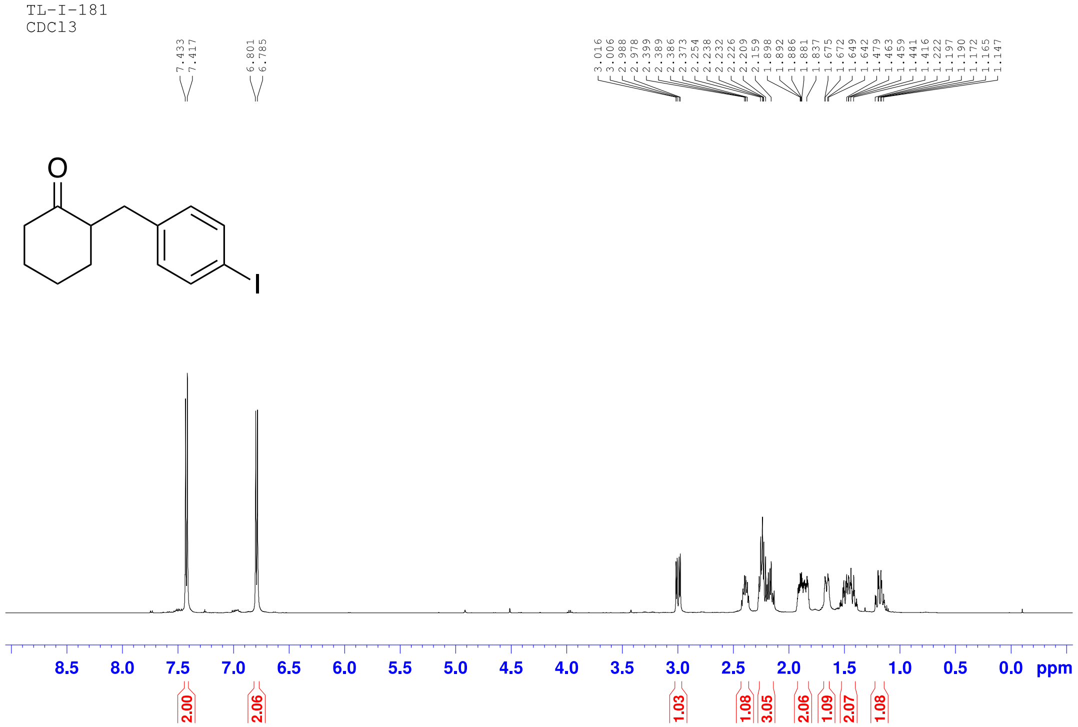
TL-I-181
CDCl3
7.433
7.417
6.801
6.785
3.016
3.006
2.988
2.978
2.399
2.389
2.386
2.373
2.254
2.238
2.232
2.226
2.209
2.159
1.898
1.892
1.886
1.881
1.837
1.675
1.672
1.649
1.642
1.479
1.463
1.459
1.441
1.416
1.222
1.197
1.190
1.172
1.165
1.147
O
I
8.5 8.0 7.5 7.0 6.5 6.0 5.5 5.0 4.5 4.0 3.5 3.0 2.5 2.0 1.5 1.0 0.5 0.0 ppm
2.00
2.06
1.03
1.08
3.05
2.06
1.09
2.07
1.08

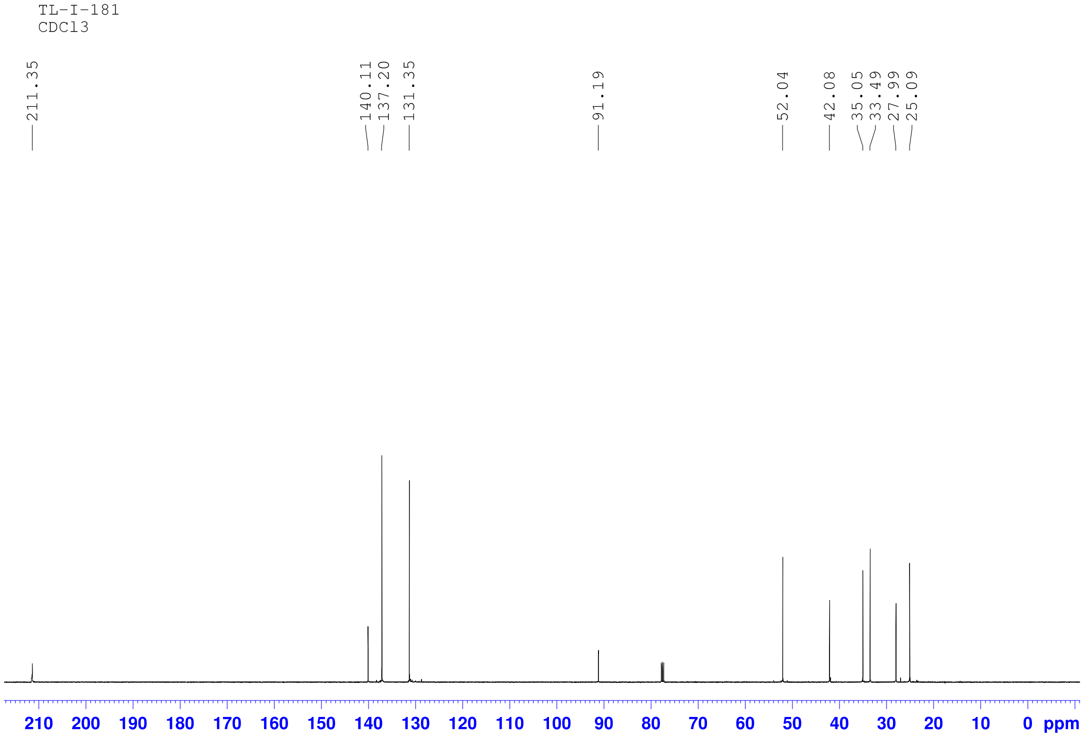
TL-I-181
CDCl3
211.35
140.11
137.20
131.35
91.19
52.04
42.08
35.05
33.49
27.99
25.09
210 200 190 180 170 160 150 140 130 120 110 100 90 80 70 60 50 40 30 20 10 0 ppm

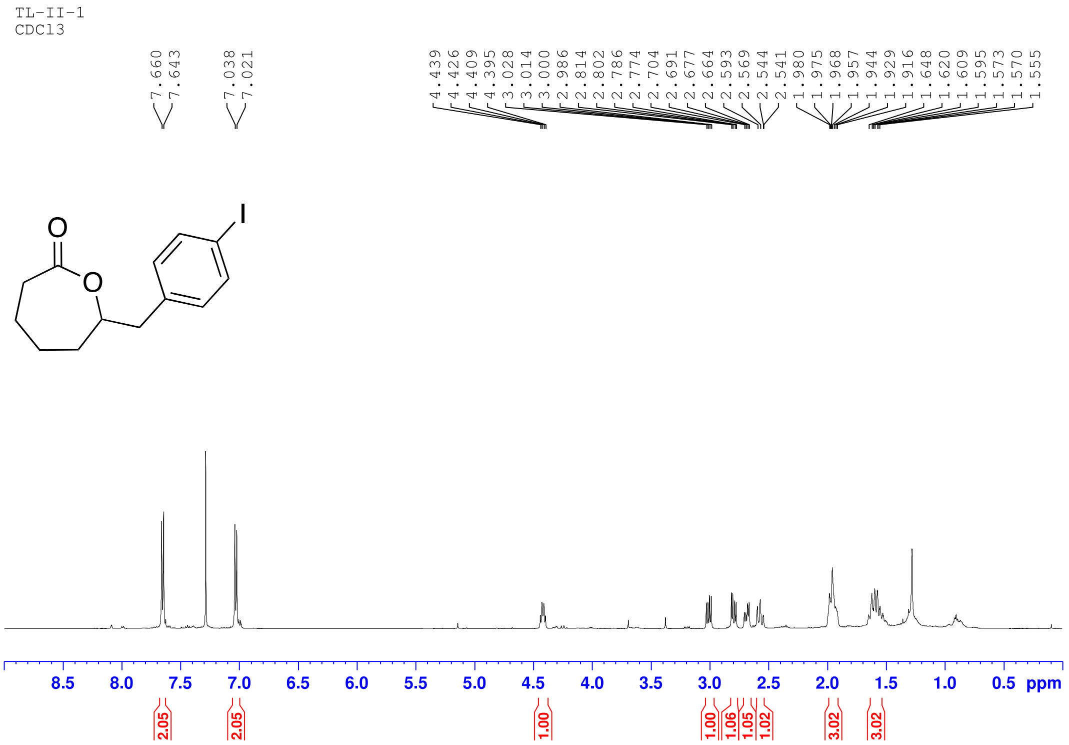
TL-II-1
CDCl3
7.660
7.643
7.038
7.021
4.439
4.426
4.409
4.395
3.028
3.014
3.000
2.986
2.814
2.802
2.786
2.774
2.704
2.691
2.677
2.664
2.593
2.569
2.544
2.541
1.980
1.975
1.968
1.957
1.944
1.929
1.916
1.648
1.620
1.609
1.595
1.573
1.570
1.555
O
O
I
8.5 8.0 7.5 7.0 6.5 6.0 5.5 5.0 4.5 4.0 3.5 3.0 2.5 2.0 1.5 1.0 0.5 ppm
2.05
2.05
1.00
1.00
1.06
1.05
1.02
3.02
3.02

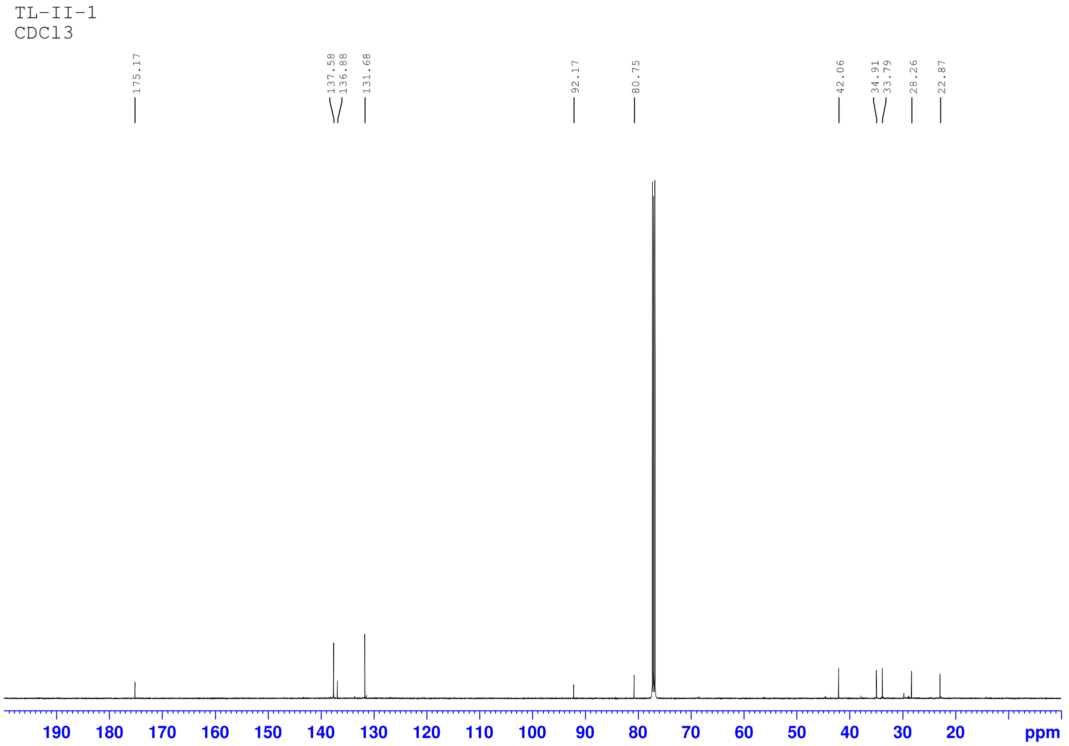
TL-II-1
CDCl3
175.17
137.58
136.88
131.68
92.17
80.75
42.06
34.91
33.79
28.26
22.87
190 180 170 160 150 140 130 120 110 100 90 80 70 60 50 40 30 20 ppm
